# *NOTCH3* T6746C and *TP53* P72R Polymorphisms Are Associated with the Susceptibility to Diffuse Cutaneous Systemic Sclerosis

**DOI:** 10.1155/2020/8465971

**Published:** 2020-02-25

**Authors:** Szymon Zmorzyński, Magdalena Wojcierowska-Litwin, Małgorzata Kowal, Małgorzata Michalska-Jakubus, Wojciech Styk, Agata Anna Filip, Irena Walecka, Dorota Krasowska

**Affiliations:** ^1^Department of Cancer Genetics with Cytogenetic Laboratory, Medical University of Lublin, Lublin, Poland; ^2^Chair and Department of Dermatology, Venerology and Pediatric Dermatology, Medical University of Lublin, Lublin, Poland; ^3^Institute of Psychology, The John Paul II Catholic University of Lublin, Lublin, Poland; ^4^Department of Dermatology, Centre of Postgraduate Medical Education, Warsaw, Poland

## Abstract

*Introduction*. NOTCH pathway and TP53 protein are involved in the development of fibrosis and autoimmune disorders, respectively. The aim of this study was to evaluate the role of single nucleotide polymorphisms (SNPs) of *NOTCH3* and *TP53* genes and serum anti-TP53 antibodies with the susceptibility, clinical subset of systemic sclerosis (SSc), and clinical profile of SSc patient, particularly with lung involvement and disease activity. *Objects and Methods*. 124 white Polish SSc patients (101 with limited cutaneous SSc–lcSSc, and 23 with diffuse cutaneous SSc–dcSSc) and 100 healthy individuals were included in the study. Patients were assessed for the presence of autoantibodies and interstitial lung disease. Two SNPs at position 6746 of *NOTCH3* and *TP53* genes and serum anti-TP53 antibodies with the susceptibility, clinical subset of systemic sclerosis (SSc), and clinical profile of SSc patient, particularly with lung involvement and disease activity.

**Results:**

The genotypic frequencies of the *NOTCH3* and *p*=0.03; *χ*^2^ = 4.63). There was no significant difference between SSc patients and the control population in allele frequencies of both SNPs. The CT + CC genotypes of *NOTCH3* and *p*=0.03; *p*=0.03; *p*=0.03; *TP53* genes and serum anti-TP53 antibodies with the susceptibility, clinical subset of systemic sclerosis (SSc), and clinical profile of SSc patient, particularly with lung involvement and disease activity. *p*=0.03;

**Conclusion:**

The CT + CC genotypes of *NOTCH3* gene and PR + RR genotypes of the *TP53* gene increased the risk of dcSSc development. Moreover, genotypes of CT + CC were associated with the active form of SSc suggesting the role of the NOTCH pathway in the pathogenesis of this disease.*NOTCH3* and *TP53* genes and serum anti-TP53 antibodies with the susceptibility, clinical subset of systemic sclerosis (SSc), and clinical profile of SSc patient, particularly with lung involvement and disease activity.

## 1. Introduction

Systemic sclerosis (SSc) is a connective tissue disease characterized by vascular dysfunction, the presence of autoantibodies, and inflammatory-driven fibrosis of the skin and internal organs [1, 2]. The disease manifests clinically as limited cutaneous SSc (lcSSc) or diffuse cutaneous SSc (dcSSc) distinguished mainly on the pattern of skin involvement: lcSSc form is characterized by skin involvement restricted to hands, face, forearms, and feet, whereas in dcSSc skin sclerosis extends proximal to the elbow and may involve truncal areas [3–5]. Interstitial lung disease is observed in up to 50% of SSc patients and is featured by activation of the NOTCH pathway involved in the differentiation of myofibroblasts [6, 7]. These cells are characterized by high proliferative capacity, which produce more extracellular matrix and in many cases do not respond to apoptotic signals [7].

NOTCH pathway is a conserved signaling system mediating cell differentiation, proliferation, survival, and apoptosis [8]. The NOTCH3 receptor regulates T-cell differentiation, which may be associated with autoimmunity [9]. *NOTCH3* gene (*locus* 19p13.12) encodes type I transmembrane receptor protein [10]. The role of single nucleotide polymorphisms (SNPs) in the coding sequence of *NOTCH3* gene remains unknown. The most common SNP, present in exon 33 (T6746C), causes a substitution of T (T allele) by C (C allele) (GTG to GCG) and results in the exchange of valine to alanine in protein chain (Val2223Ala). The residue 2223 is located in the intracellular domain, which is thought to play a role in signal transduction associated with lung fibrosis or active SSc [7]. The role of this SNP in SSc was previously not analyzed.

Auto-TP53 antibodies are detected in certain autoimmune disorders including SSc [11]. TP53 protein acts as a transcription factor, which regulates the expression of genes involved in cell cycle progression, cell growth, and apoptosis. It is encoded by the *TP53* gene (*locus* 17p13.1). The most common studied SNP (rs1042522) is located in codon 72 (exon 4) of the *TP53* gene and is associated with the presence of nucleotide with G or C (CGC to CCC). This leads to a replacement of amino acid Arg (R) with Pro (P) in protein structure [12]. The allele encoding Arg (R allele–wild type allele) was shown to induce apoptosis more effectively than the P allele [13]. Increased expression of *TP53* is consistent with a higher level of apoptosis [14].

NOTCH3 and TP53 signaling pathways are important in cell fate [15, 16]. The combination of common SNPs might influence both the susceptibility to the disease and specific features of the SSc phenotype [17].

The aim of our study was to evaluate possible associations of *NOTCH3* and *TP53* SNPs with levels of anti-TP53 antibody, clinical subsets of SSc, clinical profile of SSc patients, particular lung involvement, and disease activity.

## 2. Material and Methods

### 2.1. Patients and Samples

The study comprised 124 consecutive adult SSc patients and 100 healthy blood donors. The inclusion and exclusion criteria for SSc patients and healthy blood donors are shown in Table 1. The patients were hospitalized in the Department of Dermatology, Venerology and Pediatric Dermatology of the Medical University of Lublin between June 2017 and March 2019. All patients fulfilled the American Rheumatism Association diagnostic criteria [18, 19]. Ethical approval was obtained from the Bioethics Committee of Medical University of Lublin [KE-0254/145/2017] and each patient signed an informed consent form according to the Helsinki Declaration.

The study population was divided into two groups—lcSSc (*n* = 101) and dcSSc (*n* = 23)—according to the classification criteria for systemic sclerosis subsets [5, 20]. Furthermore, based on the disease duration from the first non-Raynaud symptom, SSc patients were divided into an early (<5 years for lcSSc and <3 years for dcSSc) and late (>5 years for lcSSc and >3 years for dcSSc) stage of SSc (*n* = 41 and *n* = 83, respectively) [21]. Routine laboratory and imaging diagnostic tests were performed to determine the disease activity and lung involvement. Levels of C-reactive protein (CRP), erythrocyte sedimentation rate (ESR), and complement components 3 and 4 (C3 and C4) were analyzed. Lung involvement was evaluated by high-resolution computed tomography (HRCT), spirometry, and diffusing lung capacity for carbon monoxide (DLCO). To determine disease activity, we used a 10-point activity index for SSc developed by the European Scleroderma Study Group (EScSG). Results of ≥3 points were indicative for active disease [22]. All patients were tested for circulating autoantibodies, such as antinuclear antibodies (ANA), anticentromere antibodies (ACA), anti-topoisomerase I (anti-Scl-70), and anti-polymerase RNAIII antibodies, following standard methods. SSc patients' characteristics are shown in Table 2.

Control peripheral blood samples were collected from 100 adult, healthy blood donors (50 males and 50 females) attending the Regional Blood Donation and Blood Treatment Center in Kielce, Poland.

### 2.2. DNA Isolation

DNA isolation from peripheral blood was performed using a commercial kit (Qiagen, Germany) according to the manufacturer's procedure. The concentration and quality of DNA were checked using NanoDrop device (Thermo Fisher Scientific, USA). A total of 124 SSc patients and 100 healthy blood donors were genotyped.

### 2.3. *NOTCH3* and *TP53* Genotyping

Two polymorphisms were assessed by PCR-restriction fragment length polymorphism (RFLP). Each PCR mix (25 *μ*l) contained 150 ng genomic DNA, PCR buffer (Clontech Laboratories, USA), dNTPs mix (0.25 mM), HD polymerase (Clontech Laboratories, USA), and primers (10 *μ*M of each). The mix was heated to 94°C for 5 min and underwent 35 cycles of amplification for *NOTCH3* and *TP53*: denaturation 98°C for 10 s and 94°C for 15 s, annealing 64°C for 10 s and 55°C for 10 s, and elongation 72°C for 20 s and 72°C for 30 s, respectively. The final elongation took 5 min at 72°C. The PCR reaction was performed using an Applied Biosystems 9700 Thermal Cycler. The following primers were used in PCR reaction:

- *NOTCH3*forward 5′-CTT ACC TGG CAG TCC CAG G-3′
- *NOTCH3*reverse 5′-AGT GGC AGT GGC TGG GCT AG-3′

or

- *TP53* forward 5′-TTG CCG TCC CAA GCA ATG GAT GA-3′
- *TP53* reverse 5′-TCT GGG AAG GGA CAG AAG ATG AC-3′

**Figure 1:**
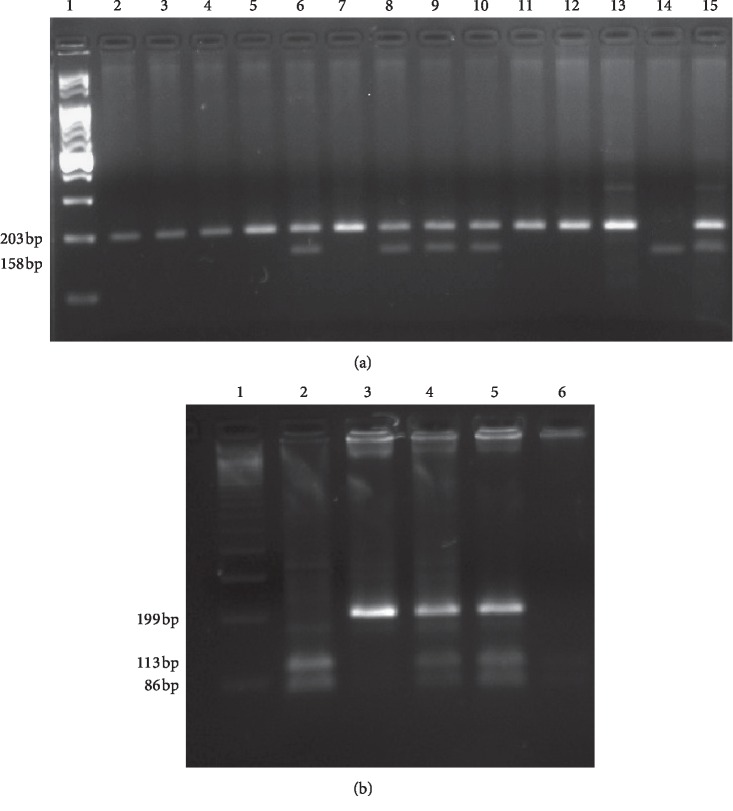
PCR-RFLP of studied polymorphisms. (a) T6746C *NOTCH3* polymorphism. Lane 1: Ladder (100 bp); Lanes 2–5, 7, and 11–13: TT genotypes; Lanes 6, 8–10, and 15: CT heterozygotes; Lane 14: CC homozygote. (b) P72R *TP53* polymorphism. Lane 1: Ladder (100 bp); Lanes 2 and 6: RR genotypes; Lane 3: PP genotype; Lanes 4 and 5: PR heterozygotes.

The PCR products of *NOTCH3* or *TP53* were digested for 16 hours at 37°C with MwoI (HpyF10VI) or *BstUI* restriction enzymes (Thermo Fisher Scientific, USA), respectively. RFLP products were analyzed on 3% agarose gel, stained with SimplySafe (Eurx, Poland), and visualized in G: Box (Syngene, Great Britain). The C or T alleles of the *NOTCH3* gene were identified by the presence of 158 bp (CC genotype) or 203 bp (TT genotypes) fragments, respectively. Heterozygous CT genotype showed the presence of two bands—158 bp and 203 bp (Figure 1(a)). The *TP53* alleles were identified by the presence of two 113 bp and 86 bp fragments (for the presence of R allele) or one fragment of 199 bp (for the presence of P allele) (Figure 1(b)). An independent PCR analysis was carried out for each sample.

**Figure 2:**
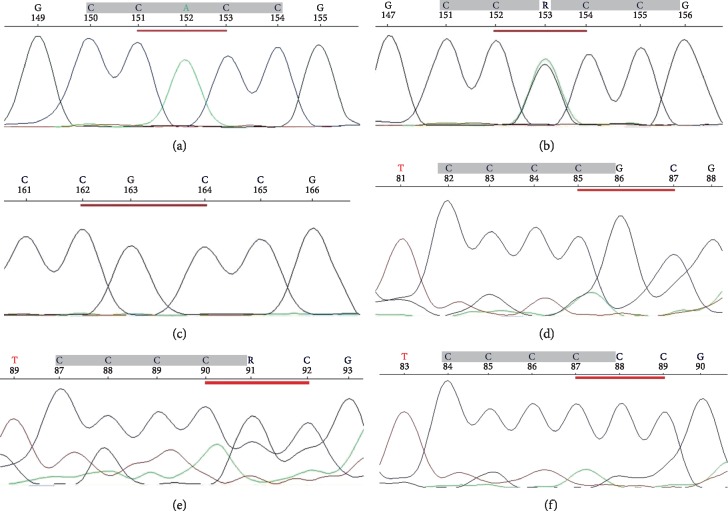
Electropherograms obtained by automated Sanger DNA sequencing. (a–c) Sequencing of the NOTCH3 gene, the sequences referred to R strand. (a) AA homozygote (on F strand–TT, T alleles), (b) heterozygote (T and C alleles), and (c) GG homozygote (on F strand–CC, C alleles). (d–f) Sequencing of the *TP53* gene. (d) GG homozygote (R alleles), (e) heterozygote (P and R alleles), and (f) CC homozygote (P alleles).

The results of *NOTCH3* and *TP53* polymorphisms analysis obtained by PCR-RFLP were confirmed by the use of automated Sanger DNA sequencing of PCR-amplicons. The same primers for PCR-RFLP were used. Each PCR mixture (25 *μ*l) contained 50 ng genomic DNA, PCR buffer (Clontech), dNTPs mixture (0.25 mM), HD polymerase (0,31U) (Clontech), and primers (10 *μ*M of each). The PCR conditions were the same as described above. Sequencing PCR was performed with the use of BigDye Terminator v3.1 Cycle Sequencing Kit (Applied Biosystems) in a thermal cycler (as previously mentioned). The sequencing PCR product was purified by the use of an exterminator kit (A&A Biotechnology). The sequencing run module was StdSeq50_POP7 in genetic analyzer 3130 (Applied Biosystems). The results were analyzed by the use of Applied Biosystems software (Figure 2).

### 2.4. Anti-TP53 Antibodies Analysis

Serum-circulating TP53 antibodies (p53 antibodies) were analyzed in all SSc groups by ELISA using a commercially available kit (Wuhan Fine Biological Technology, China) according to the manufacturer's instructions. Patients treated with steroids and/or immunosuppressive medications discontinued therapy for at least one month before sample collection. No other drugs were discontinued, on the basis of their limited impact on immune system activity.

### 2.5. Statistical Analysis

An independent *t*-test was used for the analysis of continuous variables and the Chi-square test—for categorical variables. The association of studied polymorphisms with clinical or laboratory values/factors was evaluated using the Chi-square test or Fischer's exact test (when the expected value was <5). The quantitative data was shown as frequency or percentage. Deviation of genotype frequencies in controls and cases from Hardy-Weinberg equilibrium (HWE) was assessed by the Chi-square test with one degree of freedom (d*f*) using Michael H. Court's (2005–2008) calculator [23]. For a 95% confidence interval (CI), we assumed *p*=0.05 and *χ*^2^ = 3.84; therefore, if the *χ*^2^ ≤ 3.84 and the corresponding *p* > 0.05 , then the population is in HWE. The statistical power of the study was calculated according to Bacchetti and Leung 2008 [24]. We assumed a 5% error of inference and the related level of significance *p* < 0.05 pointing to the existence of statistically significant differences. Statistical analyses were performed using the Statistica ver. 12.5 (StatSoft) software.

## 3. Results

124 SSc patients with a median age of 56.7 years (range 32–79) and 100 healthy blood donors with a median age of 34.4 years (range 18–61 years) were included in the study. Genotyping was successful in all individuals. The HWE test showed that genotypic frequencies of *NOTCH3* gene for SSc patients diverged significantly from the equilibrium, which indicates a possible association of these genotypes with the disease (Table 3). The frequencies of *TP53* genotypes were in HWE.

The differences between allele frequencies of *NOTCH3* and *TP53* genes in control and study populations were statistically insignificant (Table 4). The CT and CC genotypes of *NOTCH3* gene were analyzed as one group, because the frequency of CC homozygotes was very low in SSc patient and control groups—4% (5 cases) and 2% (2 cases), respectively. An association between CT + CC genotypes and SSc susceptibility was observed—OR (odds ratio) = 1.85, *p*=0.04 (Table 4). The statistical power of this study was 0.37. Additionally, CT + CC genotypes were associated with a 3-fold higher risk of dcSSc. The statistical power of this association was 0.66. The presence of P allele of *TP53* gene was low too, and PR and PP genotypes were analyzed in the clustered group. The association between *TP53* (PR + RR) genotypes and susceptibility of dcSSc was statistically significant (*p*=0.034) with statistical power being equal to 0.69. Furthermore, the combination effect of both studied SNPs did not show the influence on SSc susceptibility (Table 5).

Next, we analyzed the potential relationship between clinical values and laboratory results with SSc susceptibility. We observed the association of CT + CC genotypes and active form of SSc (OR = 5.46 (95% CI 2.20–13.59), *p* < 0.001 ) (Table 2) and a higher risk of dcSSc (OR = 3.43 (95% CI 0.95–12.37), *p*=0.04) (Table 4). While analyzing *TP53* genotypes, we found a relationship between PR + PP genotypes and a higher risk of dcSSc (OR = 3.30 (1.04–10.41), *p*=0.034) (Table 4).

In the case of serum level of anti-TP53 antibodies, we did not observe statistically significant differences between TT and CT + CC genotypes of *NOTCH3* gene (6.41 ng/mL versus 5.90 ng/mL (*p*=0.57)) as well as *TP53* genotypes (RR versus PR + PP) (5.54 ng/mL versus 6.67 ng/mL (*p* = 0.14)).

## 4. Discussion

In this study, we have explored the association of *NOTCH3* and *TP53* polymorphisms, as well as the level of anti-TP53 antibodies with SSc susceptibility and pattern in the Caucasian population. To our knowledge, this is the first study to elicit the significance of SNPs in SSc. Our findings suggest that the genotypes of *NOTCH3* and *TP53* genes are associated with higher susceptibility of dcSSc.

DcSSc affected various skin areas and visceral organs including lungs [25]. The development of dcSSc may be induced by cell cycle arrest, activation of DNA repair mechanisms, and inactivation of the apoptosis pathway [26]. It is known that the inhibition of apoptosis is a key mechanism of fibrosis [27]. In the present study, the relationship between PR and PP genotypes of the *TP53* gene and dcSSc susceptibility was observed. *TP53* P72R polymorphism affects the function and expression of TP53 protein. The P72R polymorphism is present in the segment encoding the transactivation domain and may increase the expression of this gene and the levels of anti-TP53 antibodies [28].

Several studies were focused on the role of serum anti-TP53 antibodies in the development of malignant disorders [29, 30]. Patients with various types of cancers and *TP53* mutations show a specific autoimmune response to TP53 protein [31]. However, some studies suggested that serum anti-TP53 antibodies might be detected in certain autoimmune disorders [32]. For example, patients with systemic lupus erythematosus secrete an antibody to the C-terminal domain of TP53, which can inhibit the function of this protein [33]. Mimura and coworkers in the study of 25 patients with SSc and 20 healthy controls found no statistical difference in the level of these antibodies between both study groups [34]. Moreover, Mahmoudi showed no significant alteration in mRNA expression levels of the *TP53* gene in fibroblasts from SSc patients compared with healthy controls [35]. This is why we did not analyze serum anti-TP53 antibody in the control population, since we focused on the association of these antibodies with SSc susceptibility and pattern: this included the levels of serum antinuclear antibodies. In our study, we did not observe a relationship between anti-TP53 antibodies level and SSc susceptibility, activity, nor interstitial lung disease.

The NOTCH pathway is involved in the pathogenesis of diseases associated with abnormal fibrosis, including the development of idiopathic pulmonary fibrosis and SSc [36–38]. Activation of the NOTCH pathway in the endothelium leads to morphological, phenotypic, and functional changes in epithelial cells [39]. Several papers suggested that an overexpression of NOTCH signaling may have fibrogenic effects in a wide spectrum of diseases, including SSc [40]. Dees et al. found the activation of the NOTCH pathway in SSc with a prominent expression of ligand Jag-1 in infiltrating T-cells [37]. In human adults, *NOTCH3* is expressed only in arterial smooth muscle cells (SMCs), and its product participates in artery maturation and specification and responses to vascular injury, regulating vascular SMCs growth and apoptosis [41]. Vascular damage is thought to be involved in SSc development [42]. Early stages of SSc are associated with reduced capillary density [43]. A possible role of *NOTCH3* in myofibroblast differentiation was postulated in an animal model with NOTCH3 synthesis inhibition [44]. In our study, we found a relationship between CT + CC genotypes with the susceptibility to various forms of SSc. These genotypes were not in HWE, which suggests their possible association with disease development.

It is known that a cross-talk exists between the NOTCH pathway and TP53 protein function. Giovannini and coworkers found, in an animal model, that activation of the Notch3 receptor might suppress the expression of p53 due to posttranscriptional mechanism [45]. However, in our study, we did not find any relationship between the combination effect of *NOTCH3, TP53* polymorphisms, and SSc risk. It is possible that these SNPs exert only a minor effect, and they are linked to other alleles.

A limitation of our study is the relatively small sample size (of lcSSc and dcSSc patients) which in part is due to the low incidence of the disease. This may bias the obtained results, which thus should be considered as preliminary to further testing. Departure from HWE, like in the case of *NOTCH3* genotype, can be indicative of potential genotyping errors, population stratification, or association to the trait [46–48]. In our study, the results obtained by the PCR-RFLP method were confirmed by automated Sanger sequencing, so we could exclude the genotyping errors. The population included in the study was ethnically homogeneous. Further analysis on a larger cohort can help to better understand the significance of *NOTCH3* and *TP53* polymorphisms in the pathobiology of SSc, including dcSSc.

## 5. Conclusions

In summary, the present study provides the first evidence that the CT + CC genotypes of *NOTCH3* gene and PR + RR genotypes of the *TP53* gene increased the risk of dcSSc development. Moreover, genotypes of CT + CC were associated with the active form of SSc suggesting the role of the NOTCH pathway in the pathogenesis of this disease.

## Figures and Tables

**Table 1:** Inclusion and exclusion criteria of SSc patients and healthy blood donors.

	Inclusion criteria	Exclusion criteria
SSc patients	(i) Adult patients (ii) Signed informed consent (iii) Diagnosed with SSc and treated in the Chair and Department of Dermatology, Venerology and Pediatric Dermatology, Medical University of Lublin, Poland (iv) Unrelated individuals (v) Caucasian race from South-Western Poland	(i) Other connective tissue diseases, SSc-like illnesses related to exposures or ingestions (ii) Non-Caucasian in race

Control group	(i) Adult, healthy blood donors (ii) Signed informed consent (iii) Agreed to have blood donated and stored for research in Regional Blood Donation and Blood Treatment Center in Kielce, Poland (iv) Caucasian race from South-Western Poland	(i) Known to be infected with HIV, syphilis, tuberculosis, hepatitis B or hepatitis C (ii) A condition in which repeated blood draws or injections pose more than minimal risk for the subject such as hemophilia, other severe coagulation disorders, or significantly impaired venous access (iii) A condition that requires active medical intervention or monitoring to avert serious danger to the participant's health or well-being (iv) Non-Caucasian in race

**Table 2:** Clinical and laboratory parameters of SSc patients according to subtypes and genotypes.

SSc features	All SSc patients *n* = 124	SSc subtypes			*NOTCH3*			*TP53*		
		lcSSc *n* = 101	dcSSc *n* = 23	*p* value	TT *n* = 97	CT + CC *n* = 27	*p* value	RR *n* = 72	PR + PP *n* = 52	*p* value
Age in years, *M*	56.7	58.96	48.26	**<0.001**	56.62	58.22	0.52	56.80	56.7	0.96
Active disease, *n* (%)	91 (73.4)	73 (72.2)	18 (78.3)	0.56	74 (76.3)	10 (37)	**<0.001**	18 (25)	15 (28.8)	0.63
Inactive disease, *n* (%)	33 (26.6)	28 (27.8)	5 (21.7)		23 (23.7)	17 (63)		54 (75)	37 (71.2)	
Disease duration in years, M	8.75	11.12	11.98	0.59	11.06	12.05	0.51	11.42	10.81	0.63
Early lcSSc, *M*	—	4.12	—	—	3.70	3.57	0.77	3.25	4.11	**0.022**
Late lcSSc, *M*	—	13	—	—	13.78	14.10	0.84	14.92	12.18	0.056
Early dcSSc, *M*	—	—	2.50	—	2.25	—	—	2.5	—	—
Late dcSSc, *M*	—	—	14.55	—	14.87	13.0	0.51	14.64	14.25	0.88

Antinuclear antibodies, *n* (%)										
Anti-Scl-70 (anti-topoisomerase I) positivity	66 (53.2)	43 (42.5)	23 (100)	**<0.001**	48 (49.5)	18 (66.7)	0.11	42 (58.4)	24 (46.1)	0.17
ACA positivity	49 (39.6)	49 (48.5)	0	**<0.001**	43 (44.3)	6 (22.3)	**0.003**	25 (34.7)	24 (46.1)	0.19
Anti-RNA polymerase III positivity	2 (1.6)	2 (2)	0	^*∗*^	2 (2)	0	^*∗*^	0	2 (3.8)	^*∗*^
Other (anti-fibrillarin, ANA-speckled pattern)	7 (5.6)	7 (7)	0	^*∗*^	4 (4.1)	3 (11)	^*∗*^	5 (6.9)	2 (3.8)	^*∗*^
Complement protein C3 level (g/L), *M*	1.23	1.14	1.08	0.38	1.11	1.17	0.34	1.09	1.15	0.20
Complement protein C4 level (g/L), *M*	0.24	0.23	0.23	0.87	0.24	0.22	0.55	0.22	0.25	0.24
C-reactive protein level (mg/L), *M*	7.75	7.67	7.93	0.93	6.93	10.54	0.21	6.45	8.58	0.34
Erythrocyte sedimentation rate (mm/h), *M*	24.0	22.55	28.30	0.15	22.49	27.66	0.17	22.21	25.36	0.33
Interstitial lung disease, *n* (%)	98 (79)	79 (78.2)	19 (82.6)	^*∗*^	76 (78.4)	22 (81.5)	0.73	55 (76.4)	43 (82.7)	0.39
Total lung capacity (TLC)—% of norm	97 (15.5)	89.2	84.35	0.31	87.47	90.26	0.40	88.94	86.62	0.55
TLC ≥ 80%	89	76	13	0.07	69	20	0.76	51	38	0.77
TLC < 80%	35	25	10		28	7		21	14	

*M*: mean. ^*∗*^Too small group for analysis.

**Table 3:** Hardy-Weinberg equilibrium for *NOTCH3* and *TP53* polymorphisms in the case and control groups according to expected E and observed O values.

	TT	CT	CC	Total	HWE *p* value and *χ*^2^^*∗*^	RR	PR	PP	Total	HWE *p* value and *χ*^2^^*∗*^
*Control*	*NOTCH3*					*TP53*				
E	67	30	3	100	*p*=0.53, *χ*^2^ = 0.38	59	36	5	100	*p*=0.63, *χ*^2^ = 0.22
O	66	32	2	100		59	35	6	100	

*Case*										
E	94	28	2	124	*p*=0.03, *χ*^2^ = 4.63	72	44	7	124	*p*=0.60, *χ*^2^ = 0.43
O	97	22	5	124		73	47	5	124	

^*∗*^If *χ*^2^ ≤ 3.84 and the corresponding *p* ≥ 0.05, then the population is in HWE.

**Table 4:** The comparison of *NOTCH3* and *TP53* allele frequencies among SSc patients and controls. The impact of studied polymorphisms on the risk of SSc.

Alleles	SSc cases, *n* = 248 (%)	Controls, *n* = 200 (%)	*p* values		
*NOTCH3*					
T	216 (87%)	164 (82%)	0.13		
C	32 (13%)	36 (18%)			
Total:	248 (100%)	200 (100%)	—		
*TP53*					
R	191 (77%)	153 (76.5%)	0.88		
P	57 (23%)	47 (23.5%)			
Total:	248 (100%)	200 (100%)	—		

Genotypes	SSc patients *n* = 124 (%)	Controls *n* = 100 (%)	OR	95% CI	*p* value

*NOTCH3*					
TT	97 (78%)	66 (66%)	Reference	—	—
CT + CC	27 (22%)	34 (34%)	1.85	1.02–3.35	**0.04**
	lcSSc *n* = 101 (%)	Controls *n* = 100 (%)			
TT	77 (76%)	66 (66%)	Reference	—	—
CT + CC	24 (24%)	34 (34%)	1.65	0.89–3.06	0.10
	dcSSC *n* = 23 (%)	Controls *n* = 100 (%)			
TT	20 (87%)	66 (66%)	Reference	—	—
CT + CC	3 (13%)	34 (34%)	3.43	0.95–12.37	**0.04**
*TP53*					
RR	72 (58%)	59 (59%)	Reference	—	—
PR + PP	52 (42%)	41 (41%)	0.96	0.56–1.64	0.88
	lcSSc *n* = 101 (%)	Controls *n* = 100 (%)			
RR	53 (52%)	59 (59%)	Reference	—	—
PR + PP	48 (48%)	41 (41%)	0.76	0.43–1.34	0.35
	dcSSC *n* = 23 (%)	Controls *n* = 100 (%)			
RR	19	59 (59%)	Reference	—	—
PR + PP	4	41 (41%)	3.30	1.04–10.41	**0.034**

**Table 5:** Combination effect of *NOTCH3* and *TP53* polymorphisms on the risk of SSc.

*NOTCH3*	*TP53*	SSc patients *n*	Controls *n*	OR	95% CI	*p* value
TT	RR	60	16	Reference	—	—
CT + CC	PR + RR	15	4	1.0	0.29–3.43	0.75

## Data Availability

The clinical data used to support the findings of this study are available from the corresponding author upon request.
